# Prioritizing vaginal hysterectomy: a stepped algorithm with vNOTES support for benign indications

**DOI:** 10.3389/fmed.2026.1771932

**Published:** 2026-02-25

**Authors:** Sezin Eda Karsli, Yusuf Ziya Kizildemir, Sibel Sak, Muhammed Erdal Sak, Hacer Uyanikoglu, Mehmet Incebiyik, Helin Kalir, Işil Işik Okuyan, Merve Civelek, Cagri Kutlugun Emral, Bekir Kahveci

**Affiliations:** 1 Department of Obstetrics and Gynecology, Şanlıurfa Training and Research Hospital, Şanlıurfa, Türkiye; 2 Department of Obstetrics and Gynecology, Faculty of Medicine, Harran University, Şanlıurfa, Türkiye; 3 Department of Nursing, Institute of Health Sciences, Harran University, Şanlıurfa, Türkiye

**Keywords:** conversion to laparoscopy, minimally invasive surgery, non-descensus uterus, surgical algorithm, vaginal hysterectomy, vNOTES

## Abstract

**Objective:**

This prospective study aimed to evaluate the clinical outcomes of a standardized surgical algorithm adopting a “vaginal-first” approach in patients with non-descensus uteri. It sought to redefine vNOTES as an “educational bridge” and a “safety valve” within this framework.

**Materials and methods:**

This single-center, prospective cohort study included 165 consecutive patients scheduled for hysterectomy for benign indications. A standardized stepwise algorithm was implemented: pure vaginal hysterectomy (VH) as the primary target; transition to vNOTES (second tier) in case of failure to progress; and laparoscopy or laparotomy as a final resort. All procedures were performed by a single surgeon.

**Results:**

Vaginal completion without abdominal incision was achieved in 157 patients (95.2%). Pure VH was successful in 87.9% (*n* = 145), while 7.3% (*n* = 12) required vNOTES support, and 4.8% (*n* = 8) were converted to laparoscopy/laparotomy. High success rates were maintained in high-risk subgroups, including obesity (93.1%) and previous cesarean sections (96.0%). Learning curve analysis showed a significant increase in pure VH rates (81.7–93.9%; *p* = 0.018) and a decrease in vNOTES utilization (11.0–3.6%; *p* = 0.045). Multivariable regression identified uterine weight as the sole independent predictor of pure VH failure.

**Conclusion:**

A “vaginal-first” approach should be the standard of care for non-descensus uteri. vNOTES functions not just as a routine method, but as an educational catalyst that enhances surgical proficiency and a critical safety valve that prevents abdominal conversion. This stepped approach optimizes resource utilization while maximizing minimally invasive benefits.

## Introduction

Hysterectomy is one of the most frequently performed major surgical procedures in gynecological practice, and the selection of the surgical route remains a subject of ongoing debate among surgeons. International guidelines, such as those from the American College of Obstetricians and Gynecologists (ACOG), emphasize that minimally invasive approaches should be preferred over abdominal hysterectomy whenever feasible, citing evidence-based advantages including reduced morbidity, faster recovery times, and cost-effectiveness. Among these approaches, vaginal hysterectomy (VH) holds the position of the “preferred approach” as the least invasive method (1, 2).

Despite these clear recommendations, the utilization rates of VH, particularly in cases without uterine prolapse (non-descensus), remain below desired levels globally. In this patient group, surgeons often gravitate towards total laparoscopic hysterectomy (TLH), which generally requires greater technological equipment and a distinct set of surgical competencies. In recent years, Vaginal Natural Orifice Transluminal Endoscopic Surgery (vNOTES) has emerged as a groundbreaking alternative for non-descensus cases by combining the advantages of endoscopic visualization and instrumentation with the vaginal route. Studies indicate that vNOTES offers significant advantages over TLH, such as shorter operative times, less postoperative pain, and faster recovery (3).

However, the growing popularity of the vNOTES technique raises an important question and a potential risk: Does this new technology cause us to overlook the true limits and potential of traditional vaginal hysterectomy? The initial and most critical stage of the vNOTES procedure essentially comprises the steps of a standard vaginal hysterectomy (4, 5). With the transection of the uterosacral ligaments, uterine mobility increases significantly; from this point, many cases can be completed without the need to transition to the endoscopic phase. This suggests that vNOTES, in experienced hands, can be positioned not as an end in itself, but as a tool providing a safe transition in challenging cases, or effectively, as a “safety valve.”

This study was designed to test a hypothesis born from this personal observation and experience. We proceeded from the premise that the process of learning the vNOTES technique serves as a “bridge” that simultaneously provides the surgeon with advanced vaginal hysterectomy experience, and as this experience grows, the need for vNOTES diminishes. The aim of this study is to present the outcomes of a stepwise surgical algorithm in patients with non-descensus uteri that targets “vaginal hysterectomy as the primary goal,” but includes a transition to vNOTES or laparoscopy when necessary. The most current systematic review and meta-analysis in this field demonstrated no significant difference between vNOTES and traditional vaginal hysterectomy regarding key surgical outcomes such as operative time, blood loss, or complication rates (6). Current evidence suggests that vNOTES does not demonstrate a distinct superiority over VH in every parameter; this situation strengthens the debate regarding why a method requiring more costly and technological equipment should be the routine first choice (6, 7). Therefore, this study aims to fill this gap in the literature by presenting the results of a stepwise surgical algorithm that integrates these two techniques rather than viewing them as rivals. In this context, we aim to redefine the role of vNOTES in modern gynecological surgery as a strategic “support mechanism.”

## Materials and methods

### Study design and ethical approval

This single-center, prospective cohort study evaluated the data of patients managed via a standardized surgical algorithm at our clinic between December 1, 2023, and October 3, 2025. The study was designed and reported in adherence to the Strengthening the Reporting of Observational Studies in Epidemiology (STROBE) guidelines. Ethical approval was obtained from the Harran University Clinical Research Ethics Committee (Date: 28.08.2023; Approval number: HRÜ/23.08.37). All data were prospectively collected and analyzed following the completion of each surgical procedure, and the study was conducted in accordance with the principles of the Declaration of Helsinki.

### Patient population and determination of surgical approach

During the study period, all patients scheduled for hysterectomy for benign gynecological indications underwent a standardized preoperative evaluation process.

#### Criteria for direct laparoscopy/laparotomy

Patients presenting with at least one of the following conditions were considered to have a primary contraindication for the vaginal route and were directed to total laparoscopic hysterectomy (TLH) or laparotomy. These patients were excluded from the analysis of the current study:

- Suspicion of malignancy or premalignant lesions during preoperative assessment.
- Uterine size evaluated as exceeding 16 gestational weeks.
- Presence of Stage 3–4 endometriosis or a history of complicated abdominal surgery suggesting extensive and severe pelvic adhesions.
- Planning of an additional procedure requiring an abdominal approach, such as sacrocolpopexy.
- Anatomical limitations that would render the initial steps of the vaginal approach impossible. This group includes populations known to present challenges for traditional VH, particularly virgo intacta transgender male patients with vaginal stenosis and atrophy due to testosterone therapy.

#### Standard algorithm cohort (study group)

All consecutive patients who did not meet the aforementioned primary exclusion criteria and had minimal or no clinically significant uterine prolapse (defined as Stage 0 or 1 according to the Pelvic Organ Prolapse Quantification [POP-Q] system) constituted the main study cohort. While Stage 1 represents a minor descent, these cases are traditionally classified as ‘non-descensus’ in surgical literature as they lack the compensatory mobility of Stage 2 or higher prolapse, thus maintaining the technical challenges of the vaginal approach. The standardized stepwise surgical algorithm defined below was applied to these patients regardless of their preoperative clinical characteristics (e.g., obesity, number of previous cesarean sections). This standardized approach was adopted to minimize surgeon-related selection bias and to objectively evaluate the real-world outcomes of the algorithm.

### Standard surgical algorithm and group definitions

For every patient in the study group, the procedure was initiated with the objective of completing a pure vaginal hysterectomy (VH). Depending on the intraoperative course, the following steps were pursued:

*Step 1 (Primary Goal)*: Pure Vaginal Hysterectomy (VH).

*Step 2 (Rescue Strategy)*: Transition to vNOTES was triggered based on specific intraoperative criteria: (I) Failure to progress, defined as the inability to safely identify or secure major pedicles within 30 min of colpotomy; (II) Inadequate visualization of the adnexa when salpingo-oophorectomy was indicated; or (III) Uncontrolled venous oozing requiring endoscopic bipolar vessel sealing.

*Step 3 (Final Resort)*: Conversion to TLH or laparotomy if Step 2 was unsuccessful.

Patients were stratified into three groups for analysis based on the final surgical method completed: Group 1 (Pure VH), Group 2 (vNOTES-Assisted VH), and Group 3 (conversion to laparoscopy/laparotomy).

### Primary and secondary outcomes

The primary outcome was the “Vaginal Completion Rate,” defined as the successful removal of the uterus via the vaginal route (either pure VH or vNOTES) without abdominal conversion. Secondary outcomes included operative time, estimated blood loss (EBL), length of hospital stay, and perioperative complications (Clavien–Dindo ≥2).

### Data collection and comparative variables

Demographic, preoperative, intraoperative, and postoperative data were collected from hospital records. The following clinical characteristics, particularly those considered to potentially influence surgical difficulty, were recorded for comparison between groups:

- Body mass index (BMI, kg/m^2^)
- Number of previous cesarean sections (none, one, two or more)
- History of non-hysterectomy/non-cesarean abdominal surgery
- Ultrasonographic size of the uterus and final pathological weight (grams)

The primary surgical outcome, operative time, was defined as the total time elapsed from the initial vaginal incision to the placement of the final vaginal cuff suture. Postoperative complications were standardized according to the Clavien–Dindo Classification (Grade ≥2).

### Surgical technique: vaginal hysterectomy (with LigaSure™)

The standardized pure vaginal hysterectomy technique applied to all patients included the following steps:

All surgical procedures were performed by a single experienced surgeon who has over 5 years of clinical experience in vaginal surgery and had performed more than 200 vNOTES procedures prior to the commencement of this study to ensure technical standardization and an accurate evaluation of the learning curve. For vNOTES procedures, a V-PORT® Advanced Access Platform (Biomicro Medical, Izmir, Turkey) was utilized. Endoscopic visualization was achieved using a 5-mm, 30-degree laparoscope, and dissection was performed with standard laparoscopic graspers and a bipolar vessel sealing device (LigaSure™). Following the placement of the patient in the dorsal lithotomy position and preparation of the surgical field, the uterus was mobilized by placing tenaculums at the 6 and 12 o’clock positions of the cervix. A diluted vasopressin solution was injected submucosally into the cervical mucosa to ensure hemostasis and delineate dissection planes. The vaginal mucosa was detached via a circumferential incision around the cervix. Anterior colpotomy was completed by entering the vesicouterine space and peritoneal cavity following blunt and sharp dissection of the bladder from the cervix. Similarly, posterior colpotomy was performed by entering the pouch of Douglas and the peritoneal cavity after separating the rectum via blunt dissection following a mucosal incision in the posterior fornix. Subsequently, the hysterectomy proceeded using a vessel sealing device (LigaSure™, Medtronic) instead of the traditional clamp-cut-tie technique. The uterosacral and cardinal ligament complexes on both sides of the uterus were identified by digital palpation close to the cervix. These ligaments were sequentially coagulated and transected using the LigaSure™ device. This maneuver provided significant downward uterine mobility. Dissection continued from the caudal to cranial direction. The vascular pedicle containing the uterine arteries and veins was isolated using forceps and safely coagulated and transected with LigaSure™. Continuing dissection towards the uterine fundus, the round ligament and tubo-ovarian ligament (or infundibulopelvic ligament if salpingo-oophorectomy was planned) were sealed and transected using the same method, and the uterus was exteriorized vaginally. Following hemostasis control, the operation was terminated by closing the vaginal cuff with appropriate suture material.

### Surgical technique: transition to vNOTES and procedure

In cases where progress could not be made during pure vaginal hysterectomy or additional intervention was required (e.g., adnexal mass, salpingectomy), a transition to vNOTES was made at any stage of the procedure. Following the decision to transition, a V-PORT® Advanced Access Platform was placed into the abdominal cavity via the vaginal route. The abdominal cavity was insufflated with carbon dioxide at a pressure of 10–12 mmHg. The remaining surgical steps (e.g., adhesiolysis, salpingo-oophorectomy, completion of hysterectomy) were completed under endoscopic visualization using standard laparoscopic instruments.

### Statistical analysis

Data analysis was performed using IBM SPSS Statistics (Version 25.0). Conformity to normal distribution was assessed using the Shapiro–Wilk test, and homogeneity of variances was evaluated using Levene’s test. For the comparison of baseline demographic and perioperative variables among the three surgical outcome groups (Pure VH, vNOTES, Conversion), ANOVA (with *post-hoc* Tukey) was used for normally distributed variables, and the Kruskal–Wallis test was used for non-normally distributed variables. Chi-square or Fisher’s Exact tests were used for categorical variables (Tables 1, 2). To avoid model overfitting given the number of failure events (*n* = 20), the multivariable logistic regression for ‘Pure VH Failure’ was restricted to the two strongest clinical predictors identified in univariate analysis: Uterine Weight and History of Cesarean Section. Furthermore, a Bonferroni correction was applied to multiple comparisons in perioperative outcomes to minimize Type I error. A post-hoc power analysis was conducted to confirm the statistical robustness of the primary outcomes. Learning curve analysis was performed by chronologically dividing the cohort into two halves (First 82 vs. Last 83 cases) and comparing results via Chi-square and *T*-tests. Missing data were managed via complete-case analysis. Statistical significance was set at *p* < 0.05 for all analyses.

**Table 1:** Demographic and baseline clinical characteristics of patients according to surgical outcome groups.

Variable	Pure VH (*n* = 145)	vNOTES (*n* = 12)	Conversion (*n* = 8)	*p*-value
Age (years), mean ± SD	48.2 ± 5.1	49.1 ± 4.8	47.9 ± 5.5	0.782
BMI (kg/m^2^), mean ± SD	29.8 ± 4.2	30.5 ± 3.9	31.1 ± 4.5	0.615
Parity, median [Min–Max]	2 [0–7]	2 [0–5]	1.5 [0–4]	0.559
Menopausal status, *n* (%)				0.890
Premenopausal	102 (70.3)	9 (75.0)	6 (75.0)	
Postmenopausal	43 (29.7)	3 (25.0)	2 (25.0)	
Previous cesarean section, *n* (%)				0.911
None	78 (53.8)	7 (58.3)	5 (62.5)	
1 cesarean	45 (31.0)	3 (25.0)	2 (25.0)	
≥2 cesarean	22 (15.2)	2 (16.7)	1 (12.5)	
History of abdominal surgery, *n* (%)	32 (22.1)	3 (25.0)	2 (25.0)	0.954
Uterine weight (g), mean ± SD	185 ± 65	245 ± 80	260 ± 95	0.031
ASA score, *n* (%)				0.820
I	68 (46.9)	5 (41.7)	3 (37.5)	
II	71 (49.0)	7 (58.3)	5 (62.5)	
III	6 (4.1)	0 (0)	0 (0)	

Data are presented as mean ± standard deviation, median [range], or number (percentage). VH, vaginal hysterectomy; vNOTES, Vaginal Natural Orifice Transluminal Endoscopic Surgery; BMI: body mass index; ASA: American Society of Anesthesiologists. ^*^Statistically significant (*p* < 0.05).

**Table 2:** Perioperative and postoperative outcomes according to surgical groups.

Variable	Pure VH (*n* = 145)	vNOTES (*n* = 12)	Conversion (*n* = 8)	*p*-value
Operative time (min), mean ± SD	68 ± 14.5	92 ± 18.2	135 ± 25.1	<0.001^*^
Estimated blood loss (mL), mean ± SD	110 ± 45	165 ± 60	310 ± 115	<0.001^*^
Length of hospital stay (days), mean ± SD	1.2 ± 0.4	1.5 ± 0.6	2.8 ± 0.9	<0.001^*^
Hemoglobin drop (g/dL), mean ± SD	1.4 ± 0.5	1.8 ± 0.7	2.5 ± 0.9	<0.001^*^
Blood transfusion, *n* (%)	2 (1.4)	0 (0)	1 (12.5)	0.068
Intraoperative complications, *n* (%)	1 (0.7)	0 (0)	1 (12.5)	0.059
Postop. complications (Clavien–Dindo ≥2), *n* (%)	3 (2.1)	1 (8.3)	1 (12.5)	0.188

Data are presented as mean ± standard deviation or number (percentage). ^*^Statistically significant (*p* < 0.05).

## Results

### Patient cohort

**Figure 1:**
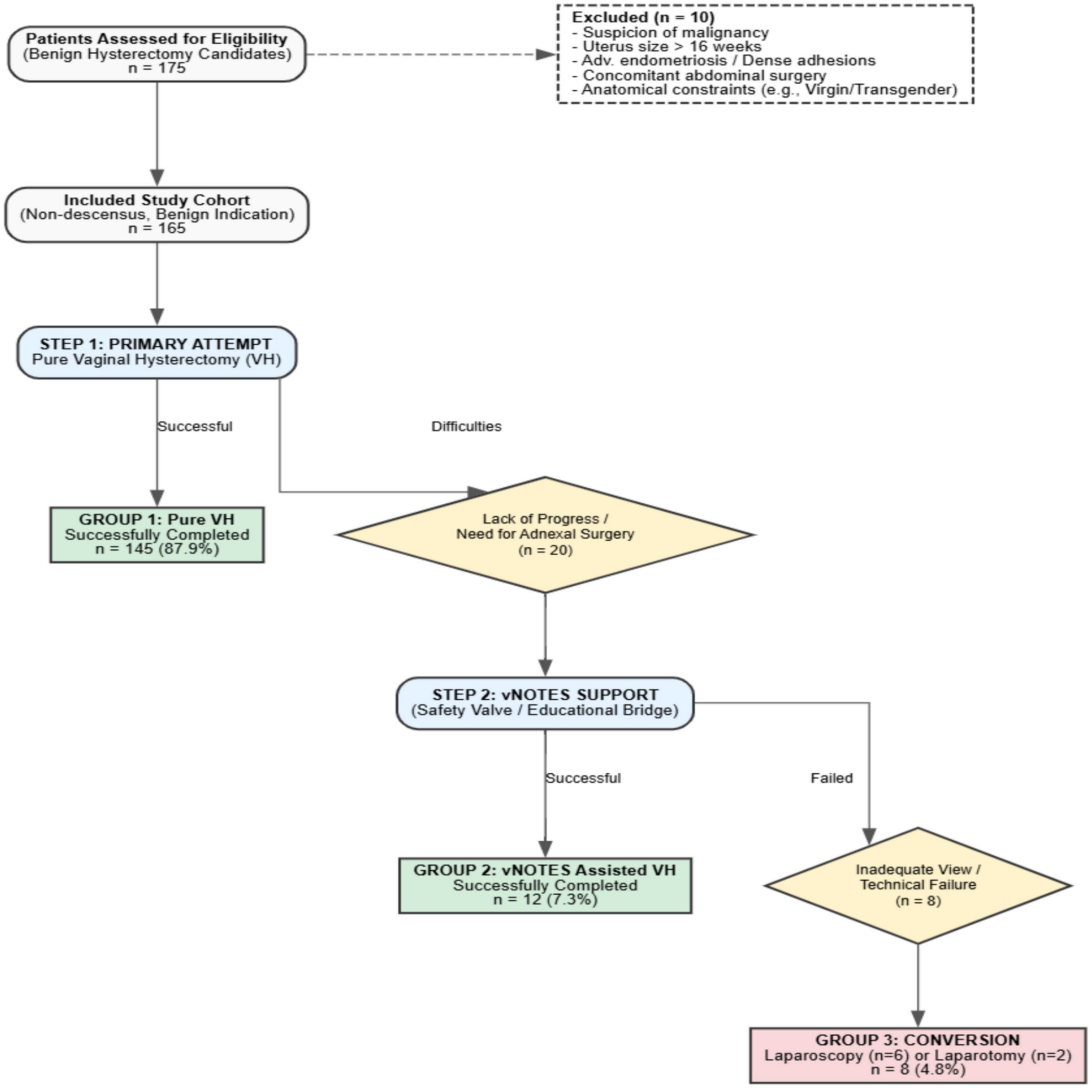
STROBE flow diagram illustrating patient selection, exclusion criteria, the stepwise surgical algorithm, and final distribution of the study groups.

During the study period, a total of 175 patients scheduled for hysterectomy for benign indications were evaluated at our clinic. Due to the primary contraindications specified in the Materials and methods section, 10 patients (5.7%) were deemed unsuitable for the standard vaginal-first algorithm and were operated on directly via laparoscopy (*n* = 7) or laparotomy (*n* = 3). These patients were excluded from the study. The remaining 165 patients constituted the main analysis cohort of the study and underwent the standard stepwise surgical algorithm (Figure 1).

### Primary endpoint: surgical success rates

Among the 165 patients managed via the standard algorithm, the operation was successfully completed via the vaginal route (either pure VH or vNOTES) without abdominal incision in 157 patients (95.2%). *Post-hoc* power analysis based on the primary outcome (vaginal completion rate) demonstrated a statistical power of 0.91, confirming the adequacy of the sample size.

The distribution of surgical methods was as follows:

- *Pure Vaginal Hysterectomy (Group 1)*: in 145 patients (87.9%), the operation was completed entirely as a pure vaginal hysterectomy without the need for any other technique.
- *vNOTES-Assisted Hysterectomy (Group 2)*: vNOTES support was required in 12 patients (7.3%) to safely complete the operation.
- *Conversion to laparoscopy/laparotomy (Group 3)*: due to the insufficiency of vaginal approaches, conversion to laparoscopy (*n* = 6) or laparotomy (*n* = 2) was required in 8 patients (4.8%).

### Comparison of demographic and clinical characteristics

The baseline demographic and clinical characteristics of the study cohort are summarized in Table 1. No statistically significant differences were found among the three surgical outcome groups (Pure VH, vNOTES, Conversion) in terms of age, body mass index (BMI), parity, menopausal status, number of previous cesarean sections, or history of abdominal surgery (*p* > 0.05 for all). Only final uterine weight was found to be statistically significantly higher in the vNOTES and Conversion groups compared to the Pure VH group (*p* = 0.031).

### Comparison of perioperative outcomes

Key surgical and postoperative outcomes are compared between groups in Table 2. As expected, operative time, estimated blood loss, and length of hospital stay significantly increased from the Pure VH group to the Conversion group (*p* < 0.001 for all). The rates of severe intraoperative and postoperative complications were generally low and did not show a statistically significant difference between the groups (p > 0.05).

### Reasons for transition to vNOTES and conversion

The most frequent reason for transitioning to vNOTES support was the need for intervention in adnexal pathology (50.0%) or inadequate uterine mobility/visualization (33.3%). In these cases, vNOTES served as a successful rescue strategy. For the conversion group, the predominant cause was dense pelvic adhesions (62.5%) making both vaginal and endoscopic progression impossible. As detailed in Table 3, some patients presented with multiple intraoperative challenges, such as uncontrollable bleeding and difficulty in anatomical orientation, necessitating an upgrade in surgical tier.

**Table 3:** Reasons for transition to vNOTES and conversion to laparoscopy/laparotomy.

Reason	Transition to vNOTES (*n* = 12 patients)	Conversion (*n* = 8 patients)
Dense pelvic adhesions	2 (16.7%)	5 (62.5%)
Intervention for adnexal pathology	6 (50.0%)	1 (12.5%)
Inadequate uterine mobility/visualization	4 (33.3%)	—
Uncontrollable bleeding/hemorrhage control	2 (16.7%)	2 (25.0%)
Difficulty in anatomical orientation	1 (8.3%)	1 (12.5%)

Percentages are calculated based on the total number of patients in the respective group (vNOTES *n* = 12, conversion *n* = 8). The sum of percentages may exceed 100% as some patients had multiple reasons reported.

### Detailed analysis of reasons for transition and conversion

The transparency of intraoperative decisions to transition to a higher-tier surgery is critical for understanding the clinical utility of the algorithm. Table 3 details the underlying reasons for the need for vNOTES support (Step 2) and conversion to laparotomy/laparoscopy (Step 3). The most frequent reason for transitioning to vNOTES was the need to perform a safe adnexal intervention, such as salpingo-oophorectomy or the evaluation of an adnexal mass (41.7%). In these instances, the endoscopic capability of vNOTES allowed for the safe completion of the procedure without an abdominal incision. Conversely, the most frequent cause for full conversion (Step 3) was dense pelvic adhesions (62.5%) that rendered both vaginal and endoscopic dissection impossible. It is important to note that intraoperative decisions were often multifaceted; for instance, some patients required transition due to a combination of inadequate mobility and difficulty in hemorrhage control, as reflected in the overlapping categories in Table 3.

### Surgical success in high-risk subgroups

To test the robustness of our algorithm, surgical success rates were examined in high-risk patient subgroups traditionally considered to present challenges for vaginal hysterectomy (Table 4). Even in the presence of obesity (BMI ≥ 30 kg/m^2^), a history of two or more cesarean sections, and a uterine weight exceeding 250 grams, pure vaginal hysterectomy and total vaginal completion rates remained remarkably high.

**Table 4:** Surgical success rates in selected high-risk subgroups.

Subgroup	Number of patients (*n*)	Pure VH rate (%)	Total vaginal completion rate (VH + vNOTES) (%)
Obesity (BMI ≥ 30 kg/m^2^)	72	84.7%	93.1%
History of ≥2 cesarean sections	25	84.0%	96.0%
Uterine weight >250 g	41	75.6%	87.8%

VH, vaginal hysterectomy; BMI, body mass index.

### Factors predicting failure of pure vaginal hysterectomy

Multivariable logistic regression analysis, performed to determine which patients failed pure VH and required vNOTES or conversion (*n* = 20), demonstrated that uterine weight was the sole independent and statistically significant predictor of failure (Table 5). Every 100-gram increase in uterine weight increased the probability of pure VH failure (i.e., the need for vNOTES or conversion) by 2.1 times [Adjusted Odds Ratio (aOR): 2.11; 95% confidence interval (CI): 1.34–3.32; *p* = 0.001]. Although BMI (aOR: 1.04; 95% CI: 0.91–1.19; *p* = 0.560) and history of cesarean section (aOR: 1.12 for 1 C/S; aOR: 1.35 for ≥2 C/S; *p* > 0.05) increased the risk of pure VH failure, these increases were not statistically significant.

**Table 5:** Multivariable logistic regression analysis of risk factors for pure vaginal hysterectomy failure (need for vNOTES or conversion).

Variable	Adjusted odds ratio (aOR)	95% confidence interval (CI)	*p*-value
Uterine weight (per 100 g increase)	2.11	1.34–3.32	0.001^*^
Body mass index (BMI)	01.04	0.91–1.19	0.560
Previous cesarean section			
Reference: none	1.00	—	—
1 cesarean section	1.12	0.45–2.78	0.812
≥2 cesarean sections	1.35	0.38–4.85	0.635
History of abdominal surgery	0.85	0.22–3.15	0.798

Outcome variable: pure VH failure (Group 2 + Group 3, *n* = 20) vs. Pure VH success (Group 1, *n* = 145). ^*^Statistically significant (*p* < 0.05).

### Impact of surgical experience (learning curve analysis)

Comparison of the ‘First 82’ and ‘Last 83’ groups showed no significant differences in baseline demographics, including mean age (48.1 vs. 48.3; *p* = 0.81), BMI (30.1 vs. 29.5; *p* = 0.42), and uterine weight (195 g vs. 188 g; *p* = 0.55), confirming that improved outcomes were related to surgical experience rather than case selection. To evaluate the effect of surgical experience and the standardization of the algorithm on outcomes, the patient cohort was chronologically divided. As shown in Table 6, as surgical experience increased over time: the pure vaginal hysterectomy rate increased significantly (from 81.7 to 93.9%; *p* = 0.018); the rate of transition to vNOTES (the need for the “safety valve”) decreased significantly (from 11.0 to 3.6%; *p* = 0.045); and the mean operative time for cases completed via pure VH was significantly shortened (from 75 min to 61 min; *p* < 0.001). The full conversion rate showed a decreasing trend from 7.3 to 2.4% (*p* = 0.134).

**Table 6:** Learning curve analysis: comparison of first and last period cases.

Variable	First 82 cases	Last 83 cases	*p*-value
Pure VH rate, *n* (%)	67 (81.7%)	78 (93.9%)	0.018^*^
vNOTES utilization rate, *n* (%)	9 (11.0%)	3 (3.6%)	0.045^*^
Conversion rate, *n* (%)	6 (7.3%)	2 (2.4%)	0.134
Mean operative time (pure VH), min	75 ± 16.1	61 ± 12.3	<0.001^*^

VH, vaginal hysterectomy; vNOTES, Vaginal Natural Orifice Transluminal Endoscopic Surgery. ^*^Statistically significant (*p* < 0.05).

These findings strongly support the hypothesis that vNOTES plays the role of an “educational bridge,” enhancing the surgeon’s competence in vaginal surgery, and becomes less necessary over time. Comparison of baseline demographic and clinical characteristics (age, BMI, parity, uterine weight) between the two periods revealed no statistically significant differences (*p* > 0.05 for all), indicating that the improvement in outcomes was attributable to increased surgical experience rather than a change in the patient population.

## Discussion

The most fundamental and striking finding of this study is that when a systematic surgical algorithm adopting a “vaginal-first” approach is applied in patients without uterine prolapse (non-descensus), the overwhelming majority of hysterectomies (95.2%) can be successfully completed via the vaginal route without abdominal incision. This high success rate was maintained even in patient subgroups with risk factors traditionally considered relative contraindications for vaginal hysterectomy, such as obesity, history of cesarean section, and large uterus. These results challenge established notions regarding the choice of surgical approach in non-descensus hysterectomy and offer a new perspective.

One of the primary results of our study is that in non-descensus cases, where the vaginal approach is traditionally considered difficult, operations could be completed via pure vaginal hysterectomy (VH) at a very high rate of 87.9%, without the need for additional endoscopic intervention. Literature frequently reports that VH rates in patients without prolapse remain lower depending on factors such as surgeon experience and patient selection, with a recent trend favoring laparoscopic hysterectomy (8, 9). The high success rate we achieved validates the core philosophy of our proposed stepped algorithm: knowing that they can transition to vNOTES when encountering a difficult step makes surgeons bolder in initiating cases via the vaginal route, even in borderline cases where they might initially plan laparoscopy. This “psychological safety net” effectively increases the number of cases completed without the actual need for vNOTES. Consequently, this high pure VH rate demonstrates that vNOTES serves not only as a “safety valve” but also has an indirect positive effect by increasing confidence in and the frequency of vaginal surgery.

The findings of our study gain further significance when interpreted alongside the most current comprehensive evidence, the 2024 systematic review and meta-analysis by Marchand et al. (6), comparing vNOTES and vaginal hysterectomy (VH). This meta-analysis clearly demonstrated no statistically significant difference in surgical outcomes between the two techniques. The fact that vNOTES does not offer a tangible advantage over VH in terms of reduced blood loss, shorter operative time, or fewer complications strongly supports our “vaginal-first” approach (6). While recent meta-analyses summarize the current evidence, we recognize the ongoing need for high-quality randomized controlled trials (RCTs) to definitively compare VH and vNOTES. We are aware of emerging RCT data potentially favoring vNOTES; however, our study provides a distinct perspective by suggesting that these two techniques are not necessarily rivals. Instead, we propose a clinical framework where VH remains the primary target, and vNOTES serves as a sophisticated support tool when the limits of pure vaginal surgery are reached. If there is no evidence-based superiority, the argument that pure vaginal hysterectomy which is simpler, less costly, and more widely accessible should be the default standard is strengthened. Interestingly, the ability to provide “better visualization and access to the adnexa,” cited as a potential advantage of vNOTES in the review by Marchand et al. (6), emerged as a concrete finding in our study. In our series, the most frequent reason for transitioning from pure VH to vNOTES was the need for adnexal intervention (Table 3). This indicates that while vNOTES need not be a routine procedure for every patient, it plays an invaluable role as a “safety valve” and “capability extender” in specific situations where the limits of traditional VH are reached (particularly when adnexal surgery is required). Therefore, the “no difference” conclusion of the meta-analysis forms the rational basis for our proposal of a stepped algorithm that utilizes both methods intelligently. Our study contributes uniquely to the literature by presenting a practical model of how these two techniques can be used in an integrated, complementary manner.

The central and most original argument of this study is the redefinition of the role of the vNOTES technique. While vNOTES has been presented in recent years as an alternative to laparoscopy, particularly in non-descensus cases, our findings suggest that its value lies not in routine application for every patient, but in two critical roles: as an “educational bridge” and a “safety valve.” Our learning curve analysis (Table 6) strongly supports this hypothesis. As surgical experience increased, pure vaginal hysterectomy rates increased significantly (from 81.7 to 93.9%), while the need for vNOTES (from 11.0 to 3.6%) and conversion rates markedly decreased. This strongly suggests that the practice of starting every case via the vaginal route enhances the surgeon’s mastery of vaginal anatomy and surgical competence over time, with vNOTES serving as a savior that reinforces self-confidence and intervenes in difficult moments during this process. In cases of adnexal pathology or inadequate uterine mobility (Table 3), transitioning to vNOTES rather than a full laparoscopic conversion preserved the minimally invasive character of the operation, yielding better outcomes for the patient. These findings indicate that vNOTES should be positioned not as a “primary option” in non-descensus hysterectomy, but as a “strategic tool in the service of the vaginal approach.” This paradigm shift has the potential to maximize minimally invasive benefits while simultaneously improving key parameters such as surgical education and cost-effectiveness. Our stepped algorithm presents a significant philosophical departure from the ‘vNOTES-first’ strategy proposed by Tekin et al. (10), which suggests initiating all cases with vNOTES regardless of difficulty. While Tekin et al. (10) successfully demonstrated the feasibility of vNOTES as a primary method, our study positions vNOTES as a strategic ‘catalyst’ in the surgeon’s professional development. Our algorithm does not reject vNOTES; rather, it utilizes it as a psychological safety net that encourages the surgeon to initiate more cases via the vaginal route. As proficiency grows, vNOTES acts as an educational bridge that hones vaginal surgical skills, eventually becoming less necessary for routine steps but remaining an invaluable rescue tool for challenging scenarios. By integrating vNOTES as both a catalyst and a safety valve, we ensure that the simplest, most cost-effective method (pure VH) is prioritized without sacrificing the benefits of endoscopic rescue when needed (6, 10).

One of the most notable findings of our study is the maintenance of a high vaginal completion rate of 93.1% in obesity (BMI ≥ 30), conventionally considered a relative contraindication for vaginal hysterectomy. This finding aligns strongly with the results of a large retrospective study by Ahmed et al. (11) involving 843 patients, which reported no statistically significant difference in conversion rates to abdominal hysterectomy or serious intraoperative complications between obese and non-obese women undergoing non-descensus vaginal hysterectomy (NDVH). This supports our argument that obesity is not an absolute barrier to the vaginal approach. On the other hand, Ahmed et al. (11) found significantly higher operative times and blood loss in the obese group. Our stepped algorithm offers a complementary strategy precisely at this point: technical difficulties encountered during the pure vaginal approach that have the potential to prolong surgery or increase bleeding can be managed more effectively by transitioning to the endoscopic visualization and instrumentation advantages of vNOTES. This “safety valve” allows potential difficulties to be overcome without the need for abdominal conversion, ensuring that the vaginal route remains the primary option even in obese patients. Consequently, our data and the large series by Ahmed et al. (11) demonstrate that obesity should no longer be viewed as a contraindication for NDVH. Similarly, vesicouterine adhesions that may occur due to previous cesarean sections constitute a significant barrier to the vaginal approach. In our study, achieving an exceptional vaginal completion rate of 96% even in patients with a history of two or more cesarean sections demonstrates the power of our stepped algorithm in this challenging patient group. This finding is supported by the recent study of Mollahüseyinoğlu Küllaç et al. (12) comparing vNOTES, VH, and TLH. In that study, the authors explicitly stated their preference for vNOTES over traditional VH in patients with a history of cesarean section, citing “wider visualization and clearer view of intra-abdominal adhesions” as the reason. This rationale aligns perfectly with our hypothesis regarding the “safety valve” role of vNOTES. While the study by Mollahüseyinoğlu Küllaç et al. (12) reveals a tendency among surgeons to avoid VH and gravitate towards vNOTES due to the potential risk of adhesions, our study takes this approach a step further. We strongly demonstrate that starting with pure VH the least invasive method for every patient with a history of cesarean section, and resorting to the endoscopic advantage of vNOTES only during challenging dissection moments, further expands the boundaries of minimally invasive surgery and maximizes the vaginal success rate.

### The role of uterine size: the sole independent risk factor

Our multivariable logistic regression analysis confirmed that uterine weight was the sole independent risk factor for pure vaginal hysterectomy failure (transition to vNOTES or conversion) (aOR: 2.11; *p* = 0.001). As seen in Table 4, large uteri (particularly >250 g) presented a challenge for the vaginal approach. However, this finding does not imply that large uteri cannot be removed vaginally. Indeed, the fact that 87.8% of patients with uteri weighing >250 g had their operations completed via the vaginal route demonstrates the efficacy of our algorithm in this challenging group. Our study reveals that the majority of large uterus cases (75.6%) can actually be completed with the simpler pure vaginal hysterectomy without even requiring vNOTES. This suggests that while uterine size is a difficulty factor, it is not an absolute contraindication within an algorithmic approach, and positioning vNOTES as a strategic tool that intervenes only when necessary may be the most accurate approach in terms of clinical and cost-effectiveness (13).

### Limitations of the algorithm and special patient populations

It is crucial to emphasize that our proposed “vaginal-first” approach is not a universal solution, and its applicability depends on patient anatomy. Our study cohort did not include transgender patients. A significant study by Donmez et al. (14) examined hysterectomy outcomes in virgo intacta transgender male patients. In this patient group, due to the narrow and atrophic vagina resulting from nulliparity and long-term testosterone use, even the initial steps of traditional vaginal hysterectomy are extremely difficult or impossible. In this specific and anatomically challenging population, pure VH, the first step of our algorithm, is not a suitable option. Donmez et al. (14) compared totally endoscopic vaginal NOTES hysterectomy (TVNH) with conventional laparoscopy (TLH) and demonstrated that TVNH offered significant advantages such as less postoperative pain and shorter hospital stay. These findings indicate that in special cases where vaginal access is restricted, vNOTES, which we position as “supportive,” is actually the most appropriate primary minimally invasive method. Therefore, while the results of our study are valid for the broad non-descensus patient population with suitable vaginal anatomy, we acknowledge that a “vNOTES first” approach is a more accurate strategy in special groups such as transgender men. This reinforces the principle that the surgical approach must be individualized according to the patient.

In our cohort, the overwhelming majority (87.9%) of patients with non-descensus uteri had their operations successfully completed with the simpler and cost-effective pure vaginal hysterectomy (VH). This raises the question of whether starting every patient routinely with vNOTES might impose an unnecessary technological and cost burden in the majority of cases. While the model by Tekin et al. (10) initiates every case with vNOTES, our algorithm positions vNOTES not as a “primary” method, but as an invaluable “safety valve” that activates in selected cases where pure VH is obstructed or additional maneuvers such as adnexal intervention are required. Indeed, the decrease in our vNOTES utilization rate from 11.0 to 3.6% as surgical experience increased demonstrates that this tool serves as an “educational bridge,” and the need for this “valve” diminishes as the surgeon’s competence in pure vaginal surgery grows. Consequently, while acknowledging the validity of the “vNOTES first” strategy in specific contexts, we argue that our stepped approach presents a more refined model that remains truer to the philosophy of minimally invasive surgery, encouraging the development of surgical skills while ensuring rational resource use.

At this point, it must also be emphasized that the “let’s do vNOTES for every suitable patient” mentality is not a rational approach. Our findings show that vNOTES-assisted operations and conversions result in significantly longer operative times and greater blood loss compared to pure vaginal hysterectomy (Table 2). We acknowledge that surgery time in cases requiring transition to vNOTES or conversion reflects the intraoperative decision-making process and the added complexity of the rescue steps rather than the efficiency of a single technique. Consequently, our primary time-efficiency analysis focused on Pure VH cases, where a significant reduction from 75 to 61 min (*p* < 0.001) was observed as experience grew. This distinction ensures that the surgical efficiency of the primary goal (VH) is not obscured by the inherent time penalty of rescue maneuvers in more complex cases. Furthermore, due to the specialized ports and instruments required, vNOTES is not a cost-effective method for routine use. Therefore, the stepped algorithm presented in our study offers a model that optimizes both clinical efficacy and efficient resource utilization by leveraging the technological advantage of vNOTES only when truly needed. Regarding the economic implications, although some argue that vNOTES may facilitate earlier discharge in complex cases, our algorithm achieved a mean hospital stay of 1.2 days in the Pure VH group without the need for specialized equipment. By successfully managing 87.9% of cases with pure VH, we avoided the costs of disposable ports and endoscopic instruments in the vast majority of the cohort, suggesting that prioritizing the vaginal route is not only clinically sound but also highly cost-effective.

Furthermore, the economic implications of our algorithm merit consideration. While a vNOTES port and endoscopic equipment add an estimated $350–$600 to the procedural cost, our VH-first approach utilized these resources in only 7.3% of cases. In contrast, a ‘vNOTES-first’ strategy for the entire cohort would have incurred significantly higher institutional costs without necessarily improving clinical outcomes for the 87.9% of patients who were successfully managed with pure VH. This underscores the role of vNOTES not as a routine necessity, but as a cost-effective ‘rescue tool’ that prevents more expensive abdominal conversions.

### Clinical implications and future recommendations

The results of our study hold important implications for non-descensus hysterectomy practice. In light of these findings, the following practical recommendations can be made: (1) In patients with non-descensus and benign pathology, barring a few absolute contraindications, the first surgical option should always be vaginal hysterectomy. (2) vNOTES should be integrated into resident and specialist training programs not as a primary procedure, but as an advanced skill and safety tool that enhances vaginal surgical competence. (3) In hospital resource planning, vNOTES equipment should be evaluated as a strategic resource to support the vaginal approach in selected cases, rather than for routine use in every case.

### Strengths and limitations

The primary strength of this study is its prospective design and the application of a standardized stepwise algorithm to all consecutive patients, providing high-quality real-world data and minimizing selection bias. The involvement of a single surgeon allowed for a controlled evaluation of the learning curve, removing inter-surgeon technical variability. While this ensures high internal validity, we acknowledge that it may limit the immediate generalizability of our findings to centers with different surgical volumes or expertise levels. The outcomes reported here reflect a high-volume setting; therefore, our results should be viewed as a proof-of-concept. Future multicenter studies involving surgeons with diverse experience levels are warranted to confirm the external validity and reproducibility of this stepped algorithm across different clinical environments. Furthermore, the inclusion of high-risk subgroup and learning curve analyses allowed for the evaluation of the algorithm’s effectiveness from different perspectives, demonstrating that the strategy remains robust even in challenging clinical scenarios. However, several limitations must be acknowledged. First, being a single-center study with a single surgeon may limit the generalizability of our findings to centers with different expertise levels. Second, although multivariable logistic regression showed uterine weight to be an independent predictor, unmeasured confounders such as “intraoperative surgeon decision” or “vaginal access difficulty” may have played a role in the transition between surgical tiers. Third, due to the low number of failure events (*n* = 20), the predictive model was intentionally restricted to avoid overfitting, meaning these results should be interpreted as exploratory rather than definitive.

In conclusion, this study provides robust evidence supporting the vaginal route as the primary standard of care for hysterectomy in patients with non-descensus uteri, barring specific absolute contraindications. Within this algorithmic framework, vNOTES is redefined not merely as a routine surgical method, but as a strategic “catalyst” in the surgeon’s professional development. It functions as an essential “educational bridge” that hones proficiency in traditional vaginal techniques and an invaluable “safety valve” that preserves the minimally invasive nature of the procedure during challenging intraoperative scenarios. By prioritizing the simplest and most cost-effective method pure vaginal hysterectomy and utilizing vNOTES support only when clinically indicated, this stepped approach optimizes hospital resource allocation while maximizing patient safety. Ultimately, our findings suggest that the intelligent integration of vNOTES into a “vaginal-first” algorithm can successfully reverse the global decline in vaginal surgery, proposing a new, rational paradigm for modern gynecological practice.

## Data Availability

The raw data supporting the conclusions of this article will be made available by the authors, without undue reservation.
